# A new chapter for *Forensic Sciences Research*

**DOI:** 10.1093/fsr/owaf013

**Published:** 2025-07-15

As the sun sets on one chapter and rises on another, *Forensic Sciences Research* stands at a moment of transformation, firmly rooted in its past successes, and yet reaching with confident hands towards an even more luminous future.

Since its inception, *Forensic Sciences Research* has grown into a journal of international repute, a trusted voice within the global forensic science community. It has become more than a publication; it has become a reference point—a compass—for researchers, practitioners, and academics seeking to explore the ever-evolving intersections of science, justice, and human understanding.

This ascent has been made possible through the dedicated stewardship of two esteemed publishing houses: Taylor & Francis and, more recently, the Oxford University Press. To them, we extend our deepest gratitude, for their vision, for their professionalism, and for the care with which they helped shape our journal’s identity and broaden its reach. Their contributions will forever remain part of the DNA of *Forensic Sciences Research*.

Now, as we turn the page, we do so with both humility and hope, announcing our transition to a new publishing partner—a decision guided largely by economic and structural considerations, but also by a firm belief in the promise that lies ahead. Starting next year, *Forensic Sciences Research* will be published by KeAi, Elsevier’s distinguished co-publishing partner in China. This transition is rooted in thoughtful reflection and pragmatic necessity, driven in part by a shared ambition to grow, evolve, and further enhance the journal’s global impact. This new editorial home brings with it not only efficiency and innovation, but also a profound commitment to excellence that mirrors our own. We are confident it, too, will soon be recognized among the leading names in international scholarly publishing.

This is not merely a change in address; it is a renewal of purpose.

We take this opportunity to express heartfelt appreciation to all those who have brought *Forensic Sciences Research* to where it is today: our devoted editorial board, our brilliant associate editors, and—most of all—our authors, whose research and insights continue to enrich the global forensic dialogue. You are the soul of this journal.

As we step into this new era, we do so not with uncertainty, but with anticipation, for we believe the best is yet to come. May this new chapter be marked by greater impact, deeper inquiry, and a continued commitment to scientific truth and integrity.

Let us walk forward together, with curiosity as our compass and collaboration as our guide.

The future of *Forensic Sciences Research* is unwritten—but its foundations are strong, its community vibrant, and its vision clear.

With gratitude,

Duarte Nuno Vieira

*Faculty of Medicine, University of Coimbra, Coimbra, Portugal*

E-mail: dnvieira.pt@gmail.com

Hejian Wu

*Shanghai Key Laboratory of Forensic Medicine, Shanghai Forensic Service Platform, Institute of Forensic Science, Ministry of Justice, PRC, Shanghai, China*

E-mail: wuhj@ssfjd.cn

